# *Clostridium difficile* Infection Caused by Binary Toxin–Positive Strains

**DOI:** 10.3201/eid1909.110814

**Published:** 2013-09

**Authors:** Marjolein P.M. Hensgens, Ed J. Kuijper

**Affiliations:** Leiden University Medical Center, Leiden, the Netherlands

**Keywords:** Clostridium difficile, binary toxin, mortality, bacteria

**To the Editor:** With interest, we read the article by Bacci et al. in which they conclude that *Clostridium difficile* strains containing the binary toxin gene were associated with a higher case-fatality rate after 30 days, even when the analysis was stratified for PCR ribotype (*1*). Although this was an appealing conclusion, in our opinion the study was severely limited by selection bias and confounding by underlying diseases. First, in Danish patients with a *C. difficile* infection (CDI), isolates were characterized only if they were isolated during outbreaks or from patients with severe diseases or if isolates were found to be moxifloxacin resistant. Therefore, selection bias was likely to occur. Second, adjustment for concurrent conditions was not performed. This adjustment was especially warranted because outbreaks on specific hospital wards (e.g., intensive care units) could have influenced the all-cause mortality rate. Last, the selection of specific patients and strains questions the generalizability of the authors’ conclusion.

In an approach to confirm the findings of Bacci et al. (*1*), we used data from a cohort study conducted during 2006–2009 in 13 Dutch hospitals (*2*). A total of 1,350 consecutive hospitalized patients with unformed feces and a positive *C. difficile* toxin test result were included in the study. We checked the 30-day survival for study patients in the Dutch Civil Registration System. For 626 (46%) of the patients, a *C. difficile* strain was available for PCR ribotyping and binary toxin gene characterization. Patient data (e.g., age, sex, hospitalization, and antimicrobial drug use in the 3 months before onset of diarrhea) were collected by review of electronic and paper patient charts and by contacting the treating physician. Underlying diseases present at hospital admission were classified into 7 disease categories (Table, footnote). In addition, during at least 6 months, the Charlson comorbidity index at admission was determined in 9 of the 13 hospitals (total of 357 CDI patients). Proportional hazards modeling was used for survival analysis. The Medical Review Ethics Committee of the Leiden University Medical Center approved this study

**Table:** Thirty-day mortality rates, stratified by binary toxin status, for persons with *Clostridium difficile* infection, the Netherlands, 2006–2009*

Binary toxin status of infecting strain	Absolute 30-day mortality		Relative 30-day mortality, HR (95% CI)†				
			No adjustment	Adjusted for age	Adjusted for age and concurrent condition		
	No.	% (95% CI)			Method 1	Method 2	Method 3
Positive							
027, N = 55	12	22 (13–35)	2.2 (1.2–4.2)	2.0 (1.1–3.8)	2.4 (1.1–5.5)	2.0 (0.8–5.4)	2.0 (0.7–5.5)
Non-027, N = 100	15	15 (9–23)	1.5 (0.8–2.6)	1.4 (0.8–2.5)	1.3 (0.6–2.7)	1.1 (0.5–2.8)	1.1 (0.4–2.9)
Negative, N = 471‡	50	11 (8–14)	1.0 (ref)	1.0 (ref)	1.0 (ref)	1.0 (ref)	1.0 (ref)
Unknown, N = 724§	100	14 (11–17)	1.3 (0.9–1.9)	1.3 (0.9–1.9)	1.4 (0.9–2.2)	1.5 (0.9–2.3)	1.3 (0.7–2.4)

*HR, hazard ratio; ref, reference. †In the model, age and Charlson index were added as continuous variables; all others were dichotomous. Method 1, adjusted for age and history of admissions and antimicrobial drug use in the prior 3 mos. Method 2, adjusted for age; diseases of the respiratory, digestive, circulatory, and genitourinary systems; endocrine diseases; neoplasms and other diseases; history of admissions and antimicrobial drug use in the prior 3 mos. Method 3, adjusted for age; history of admissions; antimicrobial drug use in the prior 3 mos.; and Charlson comorbidity index. ‡Binary toxin–positive non-027 strains belonged to 8 PCR ribotypes (76% type 078). §Binary toxin–negative strains belonged to 64 different PCR ribotypes (23% type 014).

During the study period, CDI was endemic in all hospitals in the cohort study (13 cases/10,000 admissions). The all-cause risk for dying within 30 days was 22% (12/55) for persons infected with binary toxin–positive 027 strains, 15% (15/100) for those infected with binary toxin–positive non-027 strains, and 11% (50/471) for those infected with binary toxin–negative strains (Table). Selection bias (e.g., by primarily characterizing isolates of patients with severe disease) was unlikely because the number of deaths among CDI patients without strain characterization (100/724 [14%]) was similar to that among patients with a characterized strain (77/626 [12%]; p = 0.41). Thirty-day mortality rates were significantly higher among patients with CDI due to type 027 strains than among patients with binary toxin–negative strains (hazard ratio [HR] 2.2); additional adjustment for age and concurrent condition(s) resulted in a relatively constant HR of 2.0–2.4. Patients with CDI due to binary toxin–positive non-027 strains did not have a substantially higher 30-day mortality rate (HR 1.5); additional adjustment for age and concurrent condition(s) lowered the HR to 1.1–1.4, depending on the method of adjustment.

In accordance with findings in the Danish study, we observed a high 30-day mortality rate among persons infected with type 027 isolates. The 30-day mortality rate was lower among persons infected with non-027 binary toxin–positive isolates, especially after correction for concurrent condition(s); however, confidence intervals overlapped with those for type 027. Therefore, we cannot statistically contradict the conclusion of Bacci et al. (*1*). Nevertheless, because mortality rates in our study among patients with non-027 type CDI strongly resembled mortality rates among patients with CDI caused by binary toxin–negative isolates and because the Danish study was prone to bias and lacked adjustment for confounding, we think that the results of Bacci et al. (*1*) should be interpreted with caution. Furthermore, a large clinical study from 2008 concluded that *C. difficile* type 078, which is the most frequently found binary toxin positive non-027 strain, was not associated with a high all-cause mortality rate (*3*). A more recent publication confirmed this finding (*4*). Therefore, in our opinion, there is currently no convincing epidemiologic proof that binary toxin is a marker for infection with virulent *C. difficile.*

## References

[1] Bacci S, Mølbak K, Kjeldsen MK, Olsen KEP. Binary toxin and death after *Clostridium difficile* infection. Emerg Infect Dis. 2011;17:976–82 . 10.3201/eid1706.101483 21749757 PMC3358205

[2] Hensgens MP, Goorhuis A, Dekkers OM, van Benthem BHB, Kuijper EJ. All-cause and disease specific mortality in hospitalized patients with *Clostridium difficile* infections; a multicenter cohort study. Clin Infect Dis. 2013 Jan 13 Epub ahead of print. 10.1093/cid/cis1209 23300235

[3] Goorhuis A, Bakker D, Corver J, Debast SB, Harmanus C, Notermans DW, Emergence of *Clostridium difficile* infection due to a new hypervirulent strain, polymerase chain reaction ribotype 078. Clin Infect Dis. 2008;47:1162–70 . 10.1086/592257 18808358

[4] Walk ST, Micic D, Jain R, Lo ES, Trivedi I, Liu EW, *Clostridium difficile* ribotype does not predict severe infection. Clin Infect Dis. 2012;55:1661–8. 10.1093/cid/cis786 22972866 PMC3501335

